# A 6-year-old boy presenting with traumatic evisceration following a bicycle handle bar injury: a case report

**DOI:** 10.4076/1757-1626-2-6315

**Published:** 2009-07-31

**Authors:** Minh Hung Nguyen, Adam Watson, Ed Wong

**Affiliations:** 1 Department of Surgery, Launceston General HospitalCharles Street, Launceston, Tasmania 7000Australia; 2 Department of Orthopaedics, Royal Hobart HospitalLiverpool St, Hobart, Tasmania 7000Australia

## Abstract

We report the case of a 6-year-old boy presenting with small bowel evisceration following a fall onto a bicycle handle-bar. His case is presented, a review of the literature performed and interesting photos pre and post-op are presented.

## Case presentation

A 6-year-old Caucasian boy was brought to the Emergency Department after falling onto his bicycle handlebar at the local skate park, suffering an evisceration injury. He had ridden his bicycle up a ramp and lost control, fell down from a height of about 1 metre onto the bicycle’s handlebar and immediately realised he had suffered a serious injury. He calmly got back on the bicycle and rode out of the park, where he collapsed at the gate after appealing for help from bystanders who then called an ambulance.

**Figure 1.**
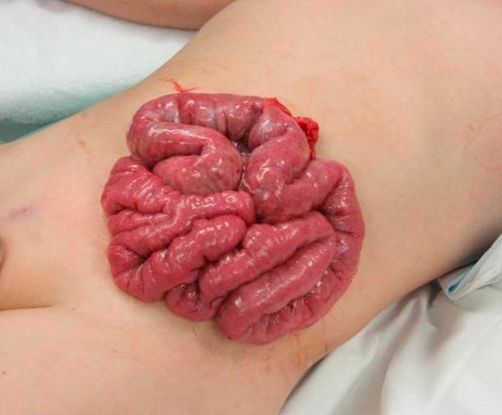
Preoperative photograph showing evisceration of small bowel.

On arrival at the Department of Accident and Emergency, he was conscious and his vital signs were stable. The obvious injury was the protrusion of the small bowels and some omentum on the abdominal wall, obscuring the wound beneath (Figure 1). History showed no previous medical history or medications of note.

After cervical and chest injuries were excluded, the resuscitated child was taken to theatre. The skin and subcutaneous tissue had been de-gloved into a 10 cm inferiorly based flap. The anterior rectus fascia was ruptured at its lateral margin in the left iliac fossa. The insertions of the external oblique, internal oblique and transversus abdominis muscles to the left rectus sheath were disrupted leaving a ragged defect 5 cm in diameter. The small bowel and a small amount of the greater omentum prolapsed through this defect. The wound did not need to be enlarged. The small bowel was not injured, nor was any other intra-abdominal organ.

**Figure 2.**
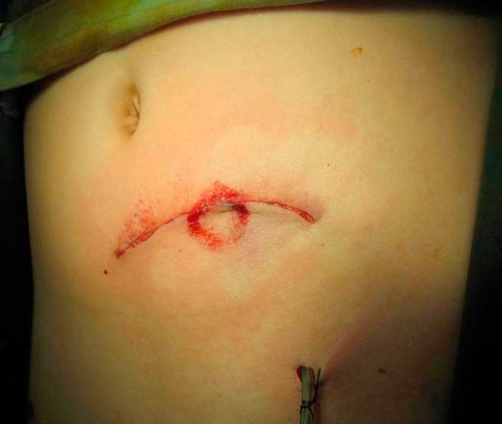
Postoperative result, revealing a circular ecchymosis at point of handlebar contact.

The bowel was inspected, cleaned and returned to the abdomen after peritoneal wash. The return was clear. The muscles and sheaths were repaired with a 1-PDS mass closure technique. The viable skin flap was heavily irrigated and repaired with a subcuticular suture over a small suction drain. (Figure 2), taken immediately after the operation, demonstrates a circular handlebar ecchymosis around the wound. The child made an uneventful recovery and was discharged after 4 days in hospital. He went back to school after two weeks.

## Discussion

Most instances of eviscerations encountered in trauma are caused by abdominal stab wound injuries, which generally requires laparotomy [1]. Evisceration following blunt abdominal wall injury, however, is less common. There have been reports of blunt evisceration following a motor vehicle accident [2]. Evisceration can occur through natural orifices, peri-anally [3] or trans-anally [4] as a result of high suction at a pool, trans-anally from an abdominal crush injury [5], trans-vaginally from a water-slide injury [6]. One report describes it occurring at a full-thickness burn of the abdominal skin overlying a paraumbilical hernia [7]. One case of evisceration occurred from a mountain bike accident and was associated with pancreatic transection [8]. Most of these eviscerations have been in young adults and children.

Handlebar injuries to the abdomen from bicycle accidents are commonly seen in emergency departments but uncommonly result in rupture of the abdominal wall, as most sole bicycle accidents occur at low speed. When a rupture of abdominal muscle occurs, the skin remains intact most of the time, and only a herniation results. A report and review of 21 reported cases of closed abdominal herniation from handlebar injuries was published in 2004 [9]. Abdominal evisceration following handle bar injury has been reported only once in 1992 [8].

Although a handle bar can act as a sharp “spear”, the wound in this case was a raised flap, visibly resulting from the handle bar impact and, as such, is better considered a blunt rather than penetrating injury. The lateral part of the lower rectus sheath is where the abdominal wall is least protected by muscles and is the site of this evisceration. This is the location of a Spigelian hernia and a common site for closed abdominal herniation secondary to trauma. In our case and that presented by Lovell [8], the bowel was not damaged. This contrasts with perineal eviscerations from pool high-suction drains, where extensive damage the bowels occurred, requiring resection [3,4].

The treatment of this injury is no different from other traumatic eviscerations. Prompt resuscitation, exclusion of other injuries, followed by early and careful laparotomy will allow the patients the best opportunity to make an uneventful recovery.

There has been a proliferation of skate parks in Australia in recent years, as local councils try to separate bicyclists and skateboarders from cars and pedestrians. These parks are seen as good places to practice airborne bicycle tricks, potentially resulting in serious injuries.
